# Family history of prostate and colorectal cancer and risk of colorectal cancer in the Women’s health initiative

**DOI:** 10.1186/s12885-017-3873-5

**Published:** 2017-12-13

**Authors:** Jennifer L. Beebe-Dimmer, Cecilia Yee, Electra Paskett, Ann G. Schwartz, Dorothy Lane, Nynikka R. A. Palmer, Cathryn H. Bock, Rami Nassir, Michael S. Simon

**Affiliations:** 1 Barbara Ann Karmanos Cancer Institute, Detroit, Michigan 48201 USA; 2 Department of Oncology, Wayne State University School of Medicine, Detroit, Michigan 48201 USA; 3 Ohio State University Comprehensive Cancer Center, Columbus, OH 43210 USA; 4 Department of Internal Medicine, School of Medicine, Columbus, OH 43210 USA; 5 Department of Preventive Medicine, Stony Brook University School of Medicine, Stony Brook, New York, 11794 USA; 6 Department of Medicine, University of California-San Francisco, San Francisco, California, 94110 USA; 7 Department of Biochemistry and Molecular Medicine, University of California-Davis, Davis, California, 95616 USA

## Abstract

**Background:**

Evidence suggests that risk of colorectal and prostate cancer is increased among those with a family history of the same disease, particularly among first-degree relatives. However, the aggregation of colorectal and prostate cancer within families has not been well investigated.

**Methods:**

Analyses were conducted among participants of the Women’s Health Initiative (WHI) observational cohort, free of cancer at the baseline examination. Subjects were followed for colorectal cancer through August 31st, 2009. A Cox-proportional hazards regression modeling approach was used to estimate risk of colorectal cancer associated with a family history of prostate cancer, colorectal cancer and both cancers among first-degree relatives of all participants and stratified by race (African American vs. White).

**Results:**

Of 75,999 eligible participants, there were 1122 colorectal cancer cases diagnosed over the study period. A family history of prostate cancer alone was not associated with an increase in colorectal cancer risk after adjustment for confounders (aHR =0.94; 95% CI =0.76, 1.15). Separate analysis examining the joint impact, a family history of both colorectal and prostate cancer was associated with an almost 50% increase in colorectal cancer risk (aHR = 1.48; 95% CI = 1.04, 2.10), but similar to those with a family history of colorectal cancer only (95% CI = 1.31; 95% CI = 1.11, 1.54).

**Conclusions:**

Our findings suggest risk of colorectal cancer is increased similarly among women with colorectal cancer only and among those with both colorectal and prostate cancer diagnosed among first-degree family members. Future studies are needed to determine the relative contribution of genes and shared environment to the risk of both cancers.

## Background

Colorectal cancer is both the 3rd most common invasive cancer diagnosed in the United States (U.S.), and 2nd most common cause of cancer mortality with a predicted 135,430 new cases diagnosed and 50,260 deaths in 2017 [1]. Prostate cancer is the most common cancer diagnosed among U.S. men with an estimated 161,360 cases and the 2nd leading cause of cancer mortality in men with 26,730 attributed deaths [1]. A positive family history of the same cancer is an important risk factor for both cancers, particularly when it is diagnosed in a first-degree family member [2–7]. Risk increases with an increasing number of affected relatives and is inversely associated with the age at diagnosis of affected relatives [2, 5, 8–12]. It is estimated that 3–6% of colorectal cancers may be attributed to rarer familial syndromes, [13] including, but not limited to, germline mutations in *MSH2*, *MSH6, MLH1*, and *PMS2* in Hereditary Non-Polyposis Colorectal Cancer (HNPCC) or Lynch syndrome, *APC* in Familial Adenomatous Polyposis (FAP) and *STK11* in Peutz-Jeghers syndrome (PJS) [14]. These syndromes carry a lifetime risk of developing colorectal cancer of up to 90% [15]. The remaining 20–30% of familial cases may be attributed to more common genes of lower penetrance, [15] potentially interacting with environmental factors. In contrast, very few genes have been consistently reported in familial and hereditary prostate cancer apart from *BRCA 1* and *BRCA2* as well as *HOXB13* [16–19]. This is despite the fact that inherited predisposition is predicted to account for 30–35% of prostate cancers [20].

Aggregation of colorectal and prostate cancer within families has not been as thoroughly investigated. Epidemiologic studies investigating the clustering of these cancers within families are conducted in populations of primarily European descent [21–26], few with an adequate number of minority patients to address racial or ethnic differences in risk associated with clustering of these cancers in families. The rationale for studies focused on clustering of these two tumors within families is partially driven by a similar underlying biology focused on exposure to adipokines (leptin and adiponectin), insulin and insulin-like growth factors, having mitogenic and potentially genotoxic effects on target tissues. The aggregation of colorectal and prostate cancer within families is likely due to a combination of both genes and shared environment, with environmental exposures occurring earlier in life perhaps more important. Similarly to our current knowledge of the contribution of genetics, a number of lifestyle and medical risk factors have been identified in colorectal cancer while very few established risk factors for prostate cancer have been identified apart from family history, age and African American race. Lastly, there are distinct racial differences in risk and survival for both cancers. African Americans are approximately 20% more likely to be diagnosed with colorectal cancer and 50% more likely to die from the disease compared to their Non-Hispanic white counterparts. Likewise, African American men are approximately 60% more likely to be diagnosed with prostate cancer and 2.5 times more likely to die compared to white men [27].

The current study evaluates the impact of a family history of prostate cancer and aggregation of prostate and colorectal cancer among first-degree relatives on risk of colorectal cancer in the Women’s Health Initiative Observational Study (WHI OS). Any evidence of clustering of these two cancers within close family members would have significant clinical implications suggesting that physicians should consider a family history of other cancers in addition to colorectal cancer and recommend earlier and more aggressive screening among women with a positive family history. Colonoscopy screening is an effective tool in reducing both colorectal cancer incidence and mortality. For individuals with a family history of colorectal cancer or adenomatous polyps in a first degree relative diagnosed before age 60 years or multiple first degree relatives diagnosed at any age (excluding suspected familial cancer syndromes), the American Cancer Society recommends colonoscopy screening to begin at age 40 or 10 years prior to the age at diagnosis of the youngest affected relative, whichever comes first and should occur every 5 years thereafter.. If family members are diagnosed after age 60 years, screening is recommended to begin at age 40 with repeat colonscopy every 10 years [28].

## Subjects and methods

The WHI consists of several clinical trials and an observational cohort with over 168,000 U.S. healthy, postmenopausal women aged 50 to 79 enrolled with active follow-up of living participants. The study details of the WHI have been previously published [29–32]. The WHI initially began as a randomized, placebo-controlled clinical trial of treatment with estrogen and progesterone to reduce the risk of coronary artery disease and a randomized, controlled clinical trial of a low-fat diet compared to a usual diet on risk of breast and colorectal cancers and coronary heart disease in postmenopausal women. Any woman who was unwilling or ineligible to participate in the clinical trials was given an opportunity to participate in the OS. Detailed information on demographics, personal medical history, and family medical history, lifestyle and behavioral risk factors was collected during a baseline interview on all OS participants.

**Fig. 1:**
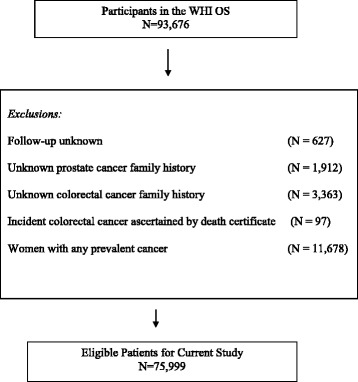
Women’s Health Initiative (WHI) Observational Study (OS)

The WHI OS study enrolled 93,676 postmenopausal women through 40 clinical centers in the United States between October 1, 1993 and December 31, 1998. The WHI OS protocol was reviewed by the Institutional Review Board at each center and informed consent was obtained from each participant locally. Each participant completed an interview and physical examination at baseline and at 3 years. Women were deemed ineligible to participate in the OS at baseline if they had a medical history which would impact participation or predicted mortality within 3 years of the baseline exam [29]. Annual questionnaires were mailed to participants to obtain follow-up data focused primarily on changes in medical history and in health behaviors. Colorectal cancers were verified using medical records and pathology was reviewed centrally by trained WHI physician adjudicators [32]. For the current study, women with any prevalent cancer at the baseline interview (*n* = 11,678), or those women whose colorectal cancer was ascertained by death certificate only (*n* = 97) were excluded (Fig. 1). In addition, we excluded women who had missing information on family history of either colorectal cancer (*n* = 3363) or prostate cancer (*n* = 1912), as well as women with an unknown period of follow-up (*n* = 627). Follow-up documentation of incident colorectal cancers was conducted through August 31st, 2009.

### Baseline data collection

At baseline, all participants had height, weight, waist and hip circumference, and blood pressure measured, and their body mass index (BMI) in kg/m^2^, calculated from measures of weight and height. Participants also completed a standardized self-administered questionnaire collecting information on demographics (including self-reported race), occupation, lifestyle risk factors for various chronic diseases (i.e., smoking, alcohol consumption and physical activity), reproductive and medical history, medication use and cancer screening behavior.

All participants were asked about their family medical history including cancer diagnoses among close relatives. The most detailed cancer family history data gathered from women were for colorectal and breast cancer, primarily due to the impact of these cancers on morbidity and mortality, as well as their inclusion as secondary end points in one or more of the clinical trial components. For both of these cancers, the number of affected first-degree relatives was recorded, the approximate age at diagnosis for each affected relative, as well as the relationship to the participant. For other cancers like prostate cancer (endometrial, cervical and ovarian cancers), only the number of affected first-degree, full-blood relatives was recorded. Data on half-siblings were not collected.

### Statistical analysis

All analyses were conducted using Statistical Analysis Systems software (SAS Inc. v.9.3, Cary, NC). Descriptive statistics were used to characterize the baseline characteristics of the study population including age, race/ethnicity, education, WHI region, BMI, waist circumference, smoking history, physical activity (in metabolic equivalent [or MET] hours per week), alcohol intake, aspirin use, hormone therapy use, insurance coverage, history of diabetes, family history of other cancers (non-colorectal, non-prostate), colorectal cancer screening within previous 5 years, and general health. Differences in the distribution of baseline characteristics between colorectal cancer cases and non-cases were evaluated separately using chi-square tests and the associated *p*-values. *P*-values of less than 0.05 were considered statistically significant. Cox proportional hazards regression was used to estimate hazard ratios (aHR) and 95% confidence intervals (CI) for colorectal cancer associated with having a family history of colorectal cancer and/or prostate cancer with adjustment for important confounders. Significant baseline characteristics were included individually in preliminary regression models. Of those characteristics, if their inclusion in the model changed the hazard ratios related to family history of either prostate or colorectal cancer by ≥10%, then these characteristics were considered important confounding variables. Models were generated for all participants combined as well as stratified by race, and for the latter analysis, participants of either non-white, non-African American were excluded. For all analyses, family history was restricted to first-degree, full blood relatives. As adjustment for some baseline characteristics such as number of first degree relatives, hormone replacement therapy use, diabetes, waist circumference, physical activity, smoking and aspirin use did not appreciably change risk estimates, final models included mutual adjustment for family history of colorectal cancer, prostate cancer, family history of other cancers, as well as age, race, and colonoscopy screening history.

## Results

Baseline characteristics of the 75,999 women included in the study are summarized in Table 1. We identified 1122 incident colorectal cancer cases during follow-up of participants with a median number of years of follow-up in the cohort of 14.6 years (InterQuartile Range = 8.5, 16.2) These cases were older at time of baseline survey (median age 66 v. 63 years; *p* < 0.0001), and were more likely than non-cases to be non-Hispanic white (85.0% v. 83.2%; *p* = 0.008), obese (28.6% v. 24.2%; *p* < 0.0001), have a greater waist circumference (*p* < 0.0001), a history of smoking (50.9% v. 47.8%; *p* = 0.04), and diabetes (7.6% v. 5.3%; 0.0008). Cases were less likely to have had a colonoscopy within 5 years of baseline interview (29.9% v. 33.5%; *p* = 0.03), and less likely to have used hormone replacement therapy (61.6% v. 69.5%; *p* < 0.0001).

**Table 1:** Baseline characteristics of Women Participating in the WHI OS

	Colorectal cancer cases		Non-cases		
Characteristic	N	(%)^a^	N	(%)^a^	*p*-value^b^
Total population	1122	1.5	74,877	98.5	
Age at baseline					
< 60	227	20.2	24,900	33.3	< 0.0001
60–69	529	47.1	32,959	44.0	
> = 70	366	32.6	17,018	22.7	
Age at diagnosis					
50–59	55	4.9	–		
60–69	296	26.4	–		
70–79	518	46.2	–		
80+	253	22.5	–		
Race/Ethnicity					0.008
Non-Hispanic White	954	85.0	62,296	83.2	
Black	98	8.7	5930	7.9	
Other	68	6.1	6449	8.6	
Unknown	2	0.2	202	0.3	
Education					0.63
No High school diploma	49	4.4	3668	4.9	
High school diploma/GED	176	15.7	12,045	16.1	
College graduate or above	890	79.3	58,560	78.2	
Unknown	7	0.6	604	0.8	
Region					0.005
Northeast	283	25.2	17,421	23.3	
South	236	21.0	19,185	25.6	
Midwest	251	22.4	16,427	21.9	
West	352	31.4	21,844	29.2	
Body mass index (kg/m^2^)					0.0003
Normal weight (<25.0)	401	35.7	30,664	41.0	
Overweight (25.0–29.9)	386	34.4	25,238	33.7	
Obese (> = 30.0)	321	28.6	18,110	24.2	
Unknown	14	1.2	865	1.2	
Waist (cm)					< 0.0001
≤ 75	210	18.7	20,114	26.9	
75.1–82.5	262	23.4	17,961	24.0	
82.6–92.5	282	25.1	18,111	24.2	
> 92.5	364	32.4	18,372	24.5	
Unknown	4	0.4	319	0.4	
Physical activity (MET-hours/week)					0.10
≤ 3.5	320	28.5	19,320	25.8	
3.5+ − 10	270	24.1	17,985	24.0	
10+ − 20	272	24.2	18,291	24.4	
> 20	249	22.2	18,551	24.8	
Unknown	11	1.0	730	1.0	
Smoking					0.04
Never	540	48.1	38,087	50.9	
Former	486	43.3	31,334	41.8	
Current	85	7.6	4529	6.0	
Unknown	11	1.0	927	1.2	
Alcohol Intake					0.05
Never/Former	318	28.3	22,090	29.5	
< 1/week or month	390	34.8	23,667	31.6	
1 - < 7/week	260	23.2	19,287	25.8	
7+/week	152	13.5	9385	12.5	
Unknown	2	0.2	448	0.6	
Aspirin^α^					0.79
Never user	720	64.2	48,194	64.4	
Inconsistent user	264	23.5	17,087	22.8	
Consistent user	138	12.3	9596	12.8	
Diabetes					0.0008
No	1036	92.3	70,837	94.6%	
Yes	85	7.6	3972	5.3%	
Unknown	1	0.1	68	0.1%	
Hysterectomy					0.91
No	677	60.3	45,265	60.5	
Yes	445	39.7	29,540	39.5	
Unknown	0	0.0	72	0.1	
Hormone Replacement Therapy Use					< 0.0001
Never	411	36.6	21,419	28.6	
Former	238	21.2	14,386	19.2	
Current	453	40.4	37,659	50.3	
Unknown	20	1.8	1413	1.9	
Insurance					0.49
No	34	3.0	2555	3.4	
Yes	1076	95.9	71,558	95.6	
Unknown	12	1.1	764	1.0	
Colonoscopy within 5 years					0.03
None done	546	48.7	34,785	46.5	
No	225	20.1	14,018	18.7	
Yes	335	29.9	25,068	33.5	
Unknown	16	1.4	1006	1.3	
Fecal occult blood test within 5 years					0.55
None done	263	23.4	16,841	22.5	
No	207	18.4	13,331	17.8	
Yes	635	56.6	43,588	58.2	
Unknown	17	1.5	1117	1.5	
Family History of Cancer^c^					0.89
No	639	57.0	42,662	57.0	
Yes	435	38.8	28,795	38.5	
Unknown	48	4.3	3420	4.6	
	Median	Range	Median	Range	
Age at baseline	66	50–79	63	49–81	
Age at diagnosis	74	52–94	–	–	
Body mass index (kg/m^2^)	26.8	15.5–66.6	26.0	11.9–69.9	

^a^Percentages may not sum to 100% due to rounding

^b^Chi-square test - excluding missing data

^c^Cancer other than colorectal or prostate among male or female relatives

α Aspirin usage: Consistent - usage of aspirin reported at both initial screening and 3-year follow-up. Inconsistent - at only one of the 2 surveys

A positive family history of colorectal cancer in a first-degree relative was associated with a 34% increase in risk of colorectal cancer among these women (aHR = 1.34; 95% CI = 1.14, 1.57) with only a marginal increase in the estimate risk when multiple affected first degree relatives were reported, but was not statistically significant (aHR = 1.40; 95% CI = 0.92, 2.11) (Table 2). Family history of prostate cancer was not associated with an increase in risk of colorectal cancer (aHR = 0.94; 95% CI = 0.76, 1.15) after controlling for colorectal cancer family history. A family history of both colorectal and prostate cancer was associated with an almost 50% increase in risk of colorectal cancer after adjustment for other important confounding factors (aHR = 1.48; 95% CI = 1.04, 2.10). Interestingly, risk of colorectal cancer in African American women with a family history of colorectal cancer appeared slightly greater (aHR = 1.80; 95% CI = 1.10, 2.93) compared with non-Hispanic white women (aHR = 1.31; 95% CI = 1.11, 1.55) (Table 3). And while there was no racial difference in colorectal cancer risk among women with a family history of prostate cancer alone (without colorectal cancer), African American women with a family history of both prostate and colorectal cancer had an approximate 75% increase in risk of colorectal cancer (aHR = 1.76; 95% CI = 0.64, 4.81), an estimate greater than for non-Hispanic white women (aHR = 1.47; 95% CI = 1.00, 2.16). No formal testing of heterogeneity by race was performed due to the relatively small number of African American cases.

**Table 2:** Baseline reported history of colorectal and prostate cancer among first-degree, full-blood relatives and colorectal cancer risk in the WHI OS

Family History of cancer among 1st degree relatives	Colorectal cancer cases		Non-cases		*p*-value	Crude HR (95% CI)	Multivariable-adjusted HR (95% CI)^a^
	N	(%)	N	(%)			
Total (75,999)	1122	1.5	74,877	98.5			
Colorectal cancer^b^					<0.001		
none	907	80.8	63,678	85.0		referent	referent
1 relative	192	17.1	10,071	13.5		1.34 (1.14–1.56)	1.34 (1.14–1.57)
> 1 relative	23	2.0	1128	1.5		1.52 (1.00–2.29)	1.40 (0.92–2.11)
Prostate cancer†					0.999		
none	1015	90.5	67,737	90.5		referent	referent
1 or more relative	107	9.5	7140	9.5		0.97 (0.80–1.19)	0.94(0.76–1.15)
Colorectal and Prostate cancer					<0.001		
None	834	74.3	58,053	77.5		referent	referent
Colorectal only	181	16.1	9684	12.9		1.30 (1.11–1.53)	1.31 (1.11–1.54)
Prostate only	73	6.5	5625	7.5		0.89 (0.70–1.13)	0.88 (0.67–1.11)
Both	34	3.0	1515	2.0		1.60 (1.14–2.26)	1.48 (1.04–2.10)

^a^Models include age, race, colonoscopy within 5 years of screening

^b^Models mutually adjust for colorectal cancer and prostate cancer family history

**Table 3:** Race-specific estimates of colorectal cancer risk associated with family history of colorectal and prostate cancer in the WHI OS

Family History of cancer among 1st degree relatives	White						Black					
	Colorectal cancer cases		Non-cases		*p*-value	Multivariable-adjusted HR (95% CI)^a^	Colorectal cancer cases		Non-cases		*p*-value	Multivariable-adjusted HR (95% CI)^a^
	N	(%)	N	(%)			N	(%)	N	(%)		
Total (78757)	954	2.0	62,296	98.0			98	2.0	5930	98.0		
Colorectal cancer†					0.001						0.033	
none	771	80.8	52,807	84.8		referent	77	78.6	5107	86.1		referent
1 or more relatives	183	19.2	9489	15.2		1.31 (1.11–1.55)	21	21.4	823	13.9		1.80 (1.10–2.93)
Prostate cancer^b^					0.805						0.576	
none	859	90.0	56,241	90.3		referent	89	90.8	5280	89.0		referent
1 or more relatives	95	10.0	6055	9.7		0.97 (0.80–1.22)	9	9.2	650	11.0		0.69 (0.33–1.42)
Colorectal and Prostate cancer					0.005						0.146	
None	705	73.9	48,010	77.1		referent	72	73.5	4612	77.8		referent
Colorectal only	154	16.1	8231	13.2		1.28 (1.07–1.52)	17	17.3	668	11.3		1.65 (0.97–2.81)
Prostate only	66	6.9	4797	7.7		0.93 (0.72–1.20)	5	5.1	495	8.3		0.51 (0.19–1.41)
Both	29	3.0	1258	2.0		1.47 (1.00–2.16)	4	4.1	155	2.6		1.76 (0.64–4.81)

^a^Models include age, colonoscopy within 5 years of baseline

^b^Models mutually adjust for colorectal and prostate cancer family history

## Discussion

Our findings suggest that a family history of prostate cancer alone is not significantly associated with risk of colorectal cancer. Although the highest risk of colorectal cancer was observed among women with a family history of both prostate and colorectal cancer, this estimate was statistically similar to the observed risk associated with having a family history of colorectal cancer only. Nevertheless, the potential for clustering of these tumors within some families have several implications: 1) Because of the known contribution of inherited predisposition for both cancers, the investigation of the clustering of these two cancers within families represents a unique framework or phenotype to identify new susceptibility genes thus contributing to our knowledge of the underlying biology of both diseases. 2) Colorectal cancer is one of the few tumors with effective screening tools that impact both primary and secondary prevention, so that identifying and screening high-risk individuals is critical in reducing both incidence and mortality. 3) It is well known that communication of colorectal cancer family history between family members is critical in risk assessment and making informed decisions about screening. However, having a complete family history of all cancers, among close relatives, even among those of the opposite sex can assist in making these decisions.

These results complement those of a recently published study examining familial clustering of breast and prostate cancer in the WHI. In this study, we observed that a family history of prostate cancer was associated with a modest increase in risk (13%) of breast cancer in the same OS cohort with the highest risk among women reporting both breast and prostate cancer diagnoses among first degree relatives. Interestingly, we examined family history of colorectal cancer and also found a marginal increase in breast cancer risk (HR = 1.08; 95% CI = 0.99–1.18) after adjustment for both breast and prostate cancer diagnosed among relatives [33]. The reverse relationship was not observed in this study, as no increase in colorectal cancer risk was observed among women with a family history of breast cancer (aHR = 1.00; 95% CI = 0.87–1.15) or women with a family history of both colorectal and breast cancer (aHR = 1.15; 95% CI = 0.85,1.54).

A family history of colorectal cancer, particularly among first-degree relatives is an established risk factor for colorectal cancer, with higher risks observed with a greater number of affected relatives, and with affected siblings (as opposed to parents) and risk inversely related to the age at diagnosis among affected relatives [2, 5, 9, 11]. A meta-analysis of 59 studies produced a pooled estimate of relative risk of 2.24 (95% CI = 2.06, 2.43) associated with having a single, first-degree relative diagnosed with colorectal cancer, while the estimate associated with having 2 or more affected first-degree relatives was 3.97 (95% CI = 2.60, 6.06) [12]. The same meta-analysis estimated the cumulative risk of developing colorectal cancer to age 70 among those with a family history (3.6% or 1 in 30), compared to the general population (1.4% or 1 in 70) with the absolute risk among individuals with a family history of colorectal cancer in multiple affected relatives increasing to 4.1% (or 1 in 24) [12]. These estimates are significantly higher than what was observed in our study. There are a couple of potential explanations for this. One explanation for this discrepancy may stem from the fact that the vast majority of studies included in the meta-analysis are case-control or cross-sectional (43 of 59) as opposed to cohort and therefore subject to different sensitivity in reporting of family history between cancer cases and controls. Furthermore, only 4 of 17 cohort studies providing data for this meta-analysis had cases which were ascertained prospectively. The remaining studies compared the incidence of colorectal cancer in relatives of colorectal cancer cases with what might be expected from the general population. The difference might also be explained by the fact that family history of cancer was assessed only at baseline in the WHI cohort, so that additional cancers diagnosed among family members post-baseline were not captured in this analysis [34].

Fewer studies have examined risk of colorectal cancer associated with a family history of other cancers including prostate cancer [21–23, 26]. An excess of endometrial cancer in Lynch syndrome families has been widely reported with a 40–60% lifetime risk of diagnosis [35]. Cancers of the stomach, small bowel, pancreas, and ovary have also been reported with less frequency in HNPCC families [14]. In a pooled analysis of case-control studies, Turati et al. observed an increased risk of colorectal cancer associated with a family history of prostate cancer that was similar to the current investigation (OR_pooled_ = 1.6) with higher estimates if the proband was diagnosed younger than age 60 (OR_pooled_ = 2.1). Similarly, an increased risk of prostate cancer was observed to be associated with a family history of colorectal cancer (OR_pooled_ = 1.5) [21]. Other studies report no significant association between colorectal cancer and family history of prostate cancer or vice-versa [3, 22–24].

To our knowledge, this is the first investigation to examine familial aggregation of colorectal and prostate cancer in a racially-diverse population and to explore the possibility that the risk relationship differs by race. The number of African American cases (*n* = 98) limited our ability to formally test for differences in estimates of risk related to family history, however our results generally suggest that African American women with a family history of colorectal cancer and of both colorectal and prostate cancer have a greater risk of being diagnosed with colorectal cancer compared with non-Hispanic white women. These findings, if replicated in a larger minority sample, are particularly important in that studies have shown that only 30–60% of individuals with a family history of colorectal cancer adhere to screening guidelines [36, 37], with some evidence to suggest that African Americans and Hispanics with a positive family history were less likely than whites to adhere to screening recommendations [36, 38].

The strengths of the current investigation include its relatively large population which allowed for precise estimation of colorectal cancer risk associated with a history of colorectal and prostate cancer among immediate family members, and particularly among those with a family history of both cancers, which is a relatively rare in the general population (~2%). Additionally, the prospective nature of the WHI cohort eliminates the potential for misclassification bias produced by differential recall of family history in colorectal cancer cases compared to non-cases. Other important strengths include the long period of follow-up for outcome with central adjudication of colorectal cancers. Study limitations include the small number of African American women with colorectal cancer in the study as well as the reliance on self-report of family history of cancer. Evidence suggests that self-reported cancer family history among first-degree relatives is generally accurate [39].

## Conclusions

In summary, family histories of both colorectal and prostate cancer and colorectal cancer only were associated with the risk of colorectal cancer in women diagnosed after age 50. However, there was no increase in risk associated with having only a family history of prostate cancer, absent colorectal cancer. There was some suggestion that African American women with a positive family history were at a greater risk compared with non-Hispanic whites, a finding deserving further study with a larger number of minorities given the racial disparities in colorectal cancer incidence and mortality.

## Abbreviations

aHR: adjusted Hazard Ratio
BMI: Body Mass Index
CI: Confidence Interval
FAP: Familial Adenomatous Polyposis
HNPCC: Hereditary Non-Polyposis Colorectal Cancer
OR: Odds Ratio
OS: Observational Study
PJS: Peutz-Jeghers syndrome
SNP: Single Nucleotide Polymorphism
WHI: Women’s Health Initiative
